# Only IF/TA in the Histological Evaluation of Post-Reperfusion Baseline Biopsies Correlates With Kidney Transplant Outcome

**DOI:** 10.3389/ti.2024.13646

**Published:** 2025-01-03

**Authors:** Quirin Bachmann, Carlos Torrez, Maike Büttner-Herold, Bernhard Haller, Flora Haberfellner, Renate Hausinger, Volker Assfalg, Lutz Renders, Kerstin Amann, Uwe Heemann, Christoph Schmaderer, Stephan Kemmner

**Affiliations:** ^1^ Department of Nephrology, University Hospital Rechts der Isar, Technical University of Munich, TUM School of Medicine and Health, Munich, Germany; ^2^ Department of Nephropathology, Institute of Pathology, Friedrich-Alexander-University Erlangen-Nuremberg (FAU) and University Hospital, Erlangen, Germany; ^3^ Institute of AI and Informatics in Medicine, University Hospital Rechts der Isar, Technical University of Munich, TUM School of Medicine and Health, Munich, Germany; ^4^ Department of Surgery, University Hospital Rechts der Isar, Technical University of Munich, TUM School of Medicine and Health, Munich, Germany; ^5^ Transplant Center, University Hospital Munich, Ludwig-Maximilians-University (LMU), Munich, Germany

**Keywords:** kidney transplantation, ischemia-reperfusion injury, delayed graft function, donor quality, interstitial fibrosis and tubular atrophy, glomerulosclerosis, arteriosclerosis, acute tubular injury

## Abstract

Here, we retrospectively evaluated the informational yield of 338 post-reperfusion kidney transplant biopsies (including 95 living donations) assessed according to BANFF for the histological characteristics interstitial fibrosis and tubular atrophy (IF/TA), glomerulosclerosis, arteriosclerosis, and acute tubular injury (ATI). Associations with delayed graft function (DGF) and death-censored graft survival were explored through Cox-regression analyses. The maximum follow-up time was 11.4 years, with DGF observed in 108 (32%) cases. After deceased donation there was no association between DGF and histologic parameters. Univariable Cox-regression unveiled an association of IF/TA and glomerulosclerosis with long-term death-censored graft survival (HR per 10% increase: IF/TA 1.63; 95% CI 1.17–2.28; *p* = 0.003; glomerulosclerosis 1.19; 95% CI 1.01–1.39; *p* = 0.031). In multivariable Cox regression analyses, adjusted for recognized clinical risk variables like expanded criteria donor-status, donor age, history of diabetes, and HLA-mismatches, only IF/TA maintained association over the total observation period in deceased donations and in the total cohort. Arteriosclerosis and ATI were not associated with clinical outcome after deceased donation. Especially ATI did not affect delayed graft function if only deceased donations were considered. Our data underlines the role of organ quality for transplant outcome prior to acute lesions such as ATI during the transplantation process.

## Graphical Abstract

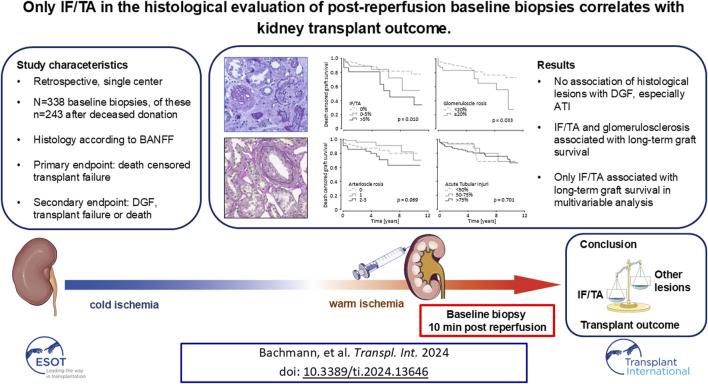

## Introduction

Kidney transplantation is the leading therapeutic option for patients with end-stage kidney disease. However, a persistent global challenge is the limited availability of donor kidneys, which fails to meet the increasing demand [1]. This discrepancy has led to an increased acceptance of kidneys from expanded criteria donors and a growing number of those allocated after rescue protocols [2–4]. However, it is noteworthy that over half of the kidneys harvested from donors aged 55 and above were not utilized in the United States as of 2021, underscoring the prevailing challenge [5, 6]. The evaluation of organ quality and the decision-making process regarding donor kidney acceptance or decline remain complex and controversial.

The utility of baseline biopsies in kidney transplantation remains unclear [7]. Baseline biopsies are routinely performed in various transplant centers at different time points, serving as valuable tools to assess graft quality and provide information about the donor kidney [8]. There are different types of baseline biopsies used in transplantation, each serving specific purposes. Procurement biopsies are employed to determine organ quality and inform decisions regarding kidney acceptance or rejection. Interestingly, procurement biopsy findings were the most common reason for discard in a retrospective analysis by Mohan et al., making them a critical factor in donor kidney allocation [9]. In contrast, pre-implantation biopsies are utilized as reference baseline biopsies for potential subsequent biopsies during the clinical follow-up. Reperfusion biopsies, taken intraoperatively after the reperfusion of the donor kidney, are also used as reference biopsies for clinical monitoring [10]. Evidence shows that punch biopsies compared to wedge biopsies are not only as save but yield higher numbers of diagnostically adequate samples according to the Banff criteria [11].

In this study, we focus on the histological finding of acute tubular injury ATI and chronic changes in post-reperfusion baseline biopsies and their potential association with long-term kidney transplant survival. We leverage a robustly characterized cohort, enabling a comparative analysis of the predictive fidelity of histologic indices against a backdrop of established clinical parameters. By elucidating these associations, we seek to compare the histological characterization of kidney grafts during the transplantation process with clinical outcome.

## Material and Methods

### Data Collection

This retrospective analysis evaluated all kidney transplantations performed between 1st January 2006, and 31st December 2016, at Klinikum rechts der Isar, Munich, Germany, both from deceased and living donors, in which a baseline biopsy was obtained during the transplant surgery via core-needle biopsy.

The analysis was approved by the local ethics committee of the Technical University of Munich, Germany (Approval No. 178/21s). Exclusion criteria were age <18 years and transplant failure due to surgical complications. Given the context in Germany where non-heart-beating kidney donation is not allowed, all deceased donations in this cohort exclusively resulted from donation after brainstem death (DBD) and will be referred to as such.

Data collection was conducted using the hospital information system, patient records, routine clinical follow-up from external nephrologists, and the Eurotransplant Network Information System - ENIS for donor and recipient data. Patient follow-up extended until 30th June 2017, which served as the data lock point.

For the subsequent statistical analysis, recipients experiencing early graft failure due to perioperative complications, including surgical and non-immunological factors, were excluded from the study.

### Endpoints

The primary endpoint of this study was death-censored transplant failure, which encompassed the permanent need for dialysis after transplantation. This includes cases of primary non-function, defined as the absence of initial allograft function with need for dialysis and without perioperative complications, confirmed by ultrasound examination showing adequate organ perfusion. Additionally, the primary endpoint also comprised cases of follow-up end-stage transplant failure, necessitating the reinstitution of dialysis. In the event of recipient death with a functioning graft, the follow-up period was censored at the date of death [12]. Patients were censored at the last day of reported kidney function during the follow-up examination within the follow up period. Primary analysis was performed including transplantations after deceased donation only. Secondary analysis included the total cohort with transplantations after deceased and living donation.

As a secondary endpoint, we considered non-death-censored transplant failure, which included a composite of primary non-function, follow-up end-stage transplant failure necessitating dialysis reinstitution, and recipient death with functioning graft.

Delayed graft function (DGF) was defined as proposed by the Organ Procurement and Transplantation Network - OPTN: need for dialysis during the first week after transplantation [13]. Recipients were subclassified whether they received an organ from standard criteria donors (SCD) or expanded criteria donors (ECD) according to the definition by Port et al. [14]. Thereby, ECDs are defined as donors who are either older than 60 years, or 50–59 years old and meet at least two of the following criteria: cerebrovascular death, history of hypertension, or last serum creatinine >1.5 mg/dL.

### Histopathology

The baseline biopsies were routinely taken 10 min after the onset of graft reperfusion using a core needle (18G) biopsy, following the clinic’s internal standard of care protocol to assess graft quality through baseline histology [15]. The samples were prepared as paraffin sections with a thickness ranging from 2 to 4 μm. These sections were then stained using hematoxylin and eosin as well as periodic acid–Schiff stains. Biopsy specimens were meticulously evaluated by an experienced renal pathologist (M.B.-H.), who remained blinded to the patients’ clinical data. All specimens were presented at the same time to decrease intra observer variability.

The degree of interstitial fibrosis and tubular atrophy (IF/TA) was reported as a percentage, representing the proportion of the affected cortical area in the biopsy sample. Severity of arteriosclerosis was evaluated using a semi-quantitative scoring system (0–3) also based on the Banff classification [16]. Glomerulosclerosis, on the other hand, was expressed as a percentage of the total number of glomeruli observed in the biopsy. The scoring of ATI was carried out following previously described criteria [15]. The assessment involved the identification of specific histologic features, such as apical blebbing, epithelial hydropic swelling with cytoplasmic lucency, loss of brush border, luminal dilatation with flattening of the epithelium, cytoplasmic vacuolization, and sloughing of tubular cells. ATI was diagnosed whenever one or more of these features were observed, and the extent of ATI was categorized as “mild” (<50%), “moderate” (50%–75%), or “severe” (>75%) tubular injury, thus generating 3 groups of comparable size.

### Statistics

Continuous data with a normal distribution are presented as mean ± standard deviation, while skewed data are summarized as median and interquartile range (IQR), represented by the first quartile to the third quartile. Categorical data are presented as absolute numbers (n) and percentages (%). Missing data was handled via available case analysis.

To compare baseline characteristics between different groups, Kruskal-Wallis and Mann-Whitney U tests were used for non-normally distributed data, univariable ANOVA and t-tests were used for normally distributed data, and chi-square (χ^2^) tests were used for categorical data. For further analysis, patients were stratified according to transplantation type (living/deceased) and according to histological outcome. ATI and arteriosclerosis were divided in groups as described above. IF/TA was analyzed in 3 groups as well: 0%, >0–5% and >5%. Glomerulosclerosis was analyzed as 2 groups: <20% and ≥20%. eGFR was then compared between histological groups at certain time points and statistical significance was calculated using Mann-Whitney U and Kruskal-Wallis test where appropriate. Patients were not included into eGFR-analysis after transplant failure and death.

Spearman rank correlation was used for associations between metric and ordinal data, and the Chi-test was used for associations between ordinal and nominal scaled variables. To assess association between histological parameters they were included into a Spearmen correlation as continuous variables (amount of change as % area), since histological outcome is not normally distributed. Mann-Whitney U and Kruskal-Wallis tests were used to compare the amount of DGF between groups. Univariable and multivariable Cox proportional-hazards models were fitted to the stratified data as described above. The Cox proportional-hazards models included recipient and donor-associated risk factors which are known to be predictive for graft survival after kidney transplantation (Table 3). IF/TA and glomerulosclerosis were included as a continuous variable in the Cox proportional-hazards analysis. For time-to-event analysis, Kaplan-Meier analysis and log-rank tests were employed to compare 1-year and long-term death censored graft survival between the histologically stratified groups for deceased donation only and the total cohort. Additionally, a multivariable Cox proportional-hazard analysis including HLA-mismatches and panel reactive antibodies (PRA) was applied to assess immunological factors in comparison to the histological outcome. Since exact timepoints of biopsy proven rejections (BPR) were not available, Spearman correlation and not Cox-analysis was used to assess association between BPRs and immunological factors. All statistical tests were performed two-sided with a significance level (α) of 0.05.

Statistical analyses were carried out using “IBM SPSS Statistics” version 29 (IBM Corp., NY, United States) and “R” version 3.4.4 (R development team, Vienna, Austria). For data visualization Adobe Illustrator, version 26.5 was utilized.

## Results

### Patients

A total of 338 kidney transplantations from living and deceased donors with baseline biopsies fulfilled the inclusion criteria for our analysis. Detailed baseline demographics are presented in Table 1.

**TABLE 1:** Demographic and clinical characteristics of donors and recipients in the total cohort and in kidney transplantations.

Characteristics	All	Living	Deceased	*p*-value
Number, n (%)	338 (100)	95	243	
Living donors, n (%)	95 (28)			
Donor				
Female, n (%)	154 (46)	55 (58)	99 (41)	0.004
Age (years)	53 ± 15	55 ± 11	52 ± 16	n.s.
BMI (kg/m^2^)	27 ± 5	27 ± 4	27 ± 5	n.s.
Cause of death (n)				
- Trauma			55 (23)	
- CVA			143 (59)	
- Other			45 (31)	
History of				
- hypertension	136 (40)	36 (38)	100 (41)	n.s.
- diabetes	32 (10)	0 (0)	32 (13)	<0.001
Last SCr (mg/dL)	0.9 [0.7; 1.1]	0.8 [0.7; 0.9]	0.9 [0.7; 1.3]	0.010
ECD	139 (41)	32 (34)	107 (44)	n.s.
Process				
HLA-Mismatch	4 [3; 5]	4 [3; 5]	4 [3; 5]	n.s.
CIT (h)	8 [2; 13]	2 [2; 2]	11 [8; 15]	<0.001
Recipient				
Female, n (%)	122 (36)	35 (37)	87 (36)	n.s.
Age (years)	52 ± 13	47 ± 13	54 ± 12	<0.001
BMI (kg/m^2^)	25 ± 5	25 ± 5	25 ± 5	n.s.
Caucasian	331 (98)	94 (99)	237 (98)	n.s.
First transplantation	282 (83)	86 (91)	196 (81)	0.028
Induction therapy	87 (26)	25 (26)	62 (26)	n.s.
Reason for ESKD				
- Glomerulonephritis	98 (29)	32 (34)	66 (27)	n.s.
- Diabetes	40 (12)	7 (7)	33 (14)	n.s.
- Hypertension	50 (15)	13 (14)	37 (15)	n.s.
- Other	150 (44)	43 (45)	107 (44)	n.s.
Dialysis vintage (months)	48 [18; 86]	3 [0; 17]	69 [38; 94]	<0.001
Immunosuppression				
- Glucocorticoids	337 (100)	95 (100)	243 (100)	n.s.
- Cni	337 (100)	95 (100)	243 (100)	n.s.
- Tacrolimus	265 (78)	89 (94)	176 (72)	<0.001
CCI	2 [2; 4]	2 [2; 3]	3 [2; 4]	0.004
kidney-pancreas transplantation	7 (2)	0 (0)	7 (3)	<0.001
Results				
Transplant failure				
- After 1 year	21 (6)	1 (1)	20 (8)	0.015 n.s.
- After 5 years	34 (10)	4 (4)	31 (13)	
- Maximum follow-up	48 (14)	7 (7)	41 (17)	0.024
Death with functioning transplant				
- After 1 year	12 (4)	1 (1)	11 (4)	0.012
- After 5 years	29 (9)	2 (2)	27 (11)	<0.001
- Maximum follow-up	38 (11)	4 (4)	34 (14)	0.001
Delayed graft function	108 (32)	12 (13)	96 (40)	<0.001
Primary non-function	12 (4)	1 (1)	11 (4)	n.s.
Patients with rejections after 1 year	93 (27)	32 (34)	61 (25)	n.s.
eGFR (ml/min/1,73 m^2^)				
- After 1 year	44 (33; 60)	50 (39; 61)	42 (32; 59)	0.032
- After 3 years	46 (36; 61)	57 (42; 68)	42 (35; 60)	0.002
Histology				
Interstitial fibrosis and tubular atrophy				n.s.
0%		64 (67)	148 (61)	
0%–5%		23 (24)	55 (23)	
>5%		8 (8)	38 (16)	
Glomerulosclerosis				0.022
<20%		84 (89)	183 (79)	
≥20%		10 (11)	50 (24)	
Arteriosclerosis grade				0.045
0		39 (44)	95 (44)	
1		33 (38)	55 (26)	
2–3		16 (18)	64 (30)	
Acute tubular injury				<0.001
<50%		48 (51)	36 (15)	
50%–75%		27 (29)	47 (20)	
>75%		19 (20)	158 (66)	

n (%) for categorical data, mean ± standard deviation for normally distributed data, median [interquartile range] for skewed data. BMI, Body Mass Index; CCI, Charlson Comorbidity; CIT, cold ischemia time; eGFR, estimated glomerular filtration rate; ECD, expanded criteria donor; ESKD, end stage kidney disease; HLA, Human leukocyte antigen; SCr, Serum creatinine. Chi-squared test was used to compare frequencies, t-test was used to compare normally distributed metric data, Mann-Whitney-U-test was used to compare nominal and not normally distributed metric data.

The median follow-up time for recipients at the time of data extraction from the clinical follow-up database was 3.4 (0.0–11.4) years. During observation, three patients were lost to follow-up and censored: one patient after deceased donation after 54 days and two patients after living donation (after 342 and 428 days). Patients without event were censored after follow-up.

### Transplant Outcomes

In the study, primary non-function (PNF) was observed in 12 (4%) of the transplantations, while DGF was experienced in 108 (32%) of the transplantations.

The median estimated glomerular filtration rate (eGFR) at various post-transplantation intervals was assessed, registering 43 [32; 54] mL/min/1.73 m^2^ at 3 months, 44 [34; 60] mL/min/1.73 m^2^ at 1 year, and escalating to 46 [36; 61] mL/min/1.73 m^2^ at the 3-year mark. Living donations presented an eGFR of 48 [36; 57] mL/min/1.73 m^2^ after 3 months, significantly higher than the 40 [30; 53] mL/min/1.73 m^2^ recorded for deceased donations (*p* = 0.017). This trend persisted, with living donations registering 50 [39; 61] mL/min/1.73 m^2^ after 1 year and 57 [42; 68] mL/min/1.73 m^2^ after 3 years, compared to 42 [32; 59] mL/min/1.73 m^2^ at 1 year and 42 [34; 60] mL/min/1.73 m^2^ and 3 years for deceased donations (Table 1).

To discern the interrelationships between histological parameters, a nonparametric correlation analysis was employed. A noteworthy observation was the minimal yet significant association between ATI and IF/TA (r = 0.11; *p* = 0.042). In alignment with anticipatory postulations, pronounced correlations were evident between IF/TA and glomerulosclerosis (r = 0.44; *p* < 0.001) and between IF/TA and arteriosclerosis (r = 0.25; *p* < 0.001). Moreover, a small but significant association was delineated between glomerulosclerosis and arteriosclerosis (r = 0.15; *p* = 0.012).

Intriguingly, a comparative evaluation between living and deceased donations revealed no significant disparities in the prevalence of IF/TA although there was a trend towards better outcomes after living donation. Glomerulosclerosis and arteriosclerosis proved to be of lower level in living donation as well. Greatest differences were observed in the incidence of ATI, which was conspicuously elevated in deceased donations (*p* < 0.001), suggesting a potential implication of the donation and preservation process on acute renal histological manifestations.

### Predictive Value of Baseline Biopsies

**FIGURE 1:**
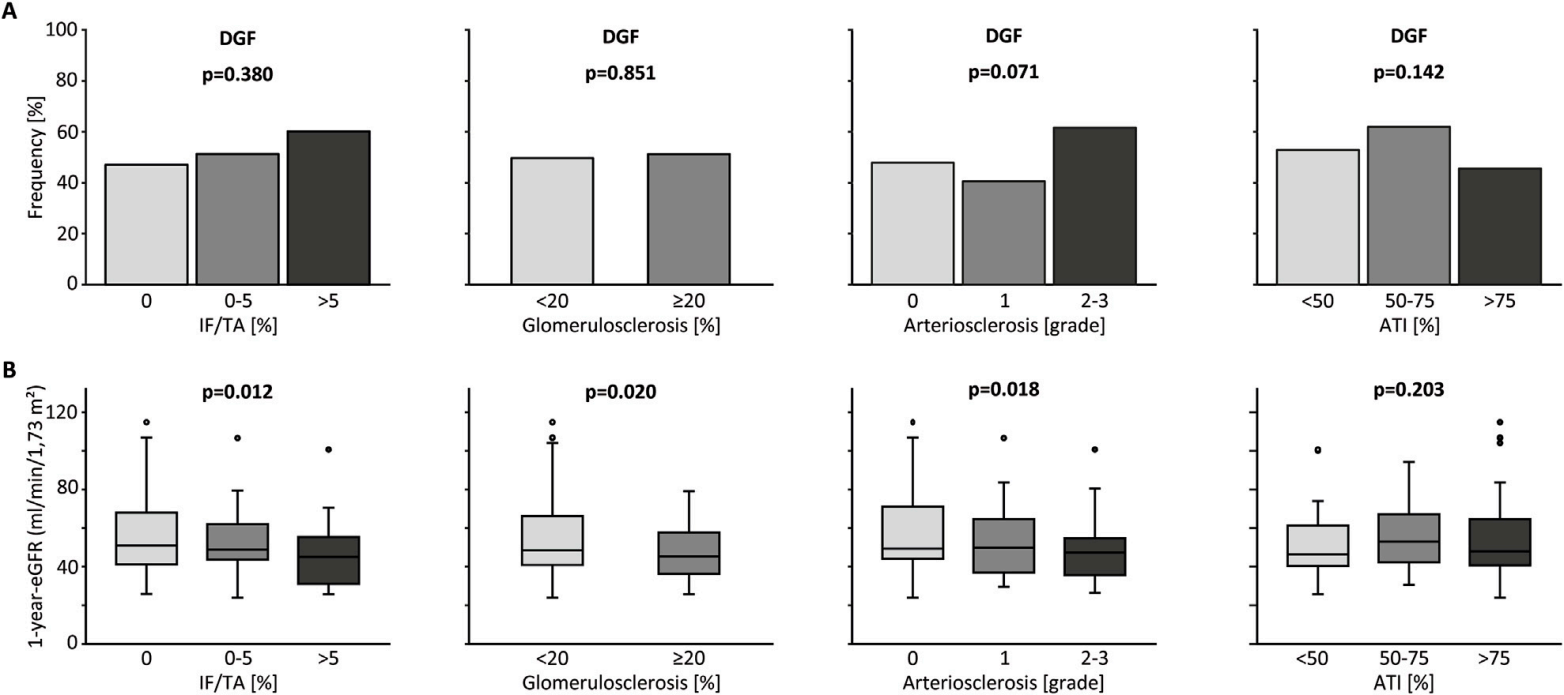
Kidney graft tissue was taken 10 min after reperfusion by 18G core needle biopsy. Histological evaluation was performed by one experienced renal pathologist. A semi-quantitative score according to the Banff Classification was used to assess arteriosclerosis (AS). Interstitial fibrosis and tubular atrophy (IF/TA), glomerulosclerosis (GS), and acute tubular injury (ATI) are shown as percentage of the entire area used for histological investigation. Only data from transplantation after deceased donation is included in this analysis. **(A)** Percent stacked column chart of the amount of delayed graft function (DGF) for different amounts of IF/TA, GS, AS, and ATI. **(B)** Boxplots of the eGFR or kidney transplant recipients after living and deceased donation 1 year after transplantation. Groups are divided by histological categories as in **(A)**. Chi-squared test was used to compare categorial data. Kruskal-Wallis test was used for comparison of >2 groups with metric variables. Mann-Whitney U test was used for comparison of 2 groups with metric variables.

When analyzing transplantations after deceased donations only, IF/TA, glomerulosclerosis and arteriosclerosis did not significantly impact the amount of DGF, although a trend towards higher rates of DGF with increasing histological damage was visible (Figure 1A). Surprisingly, there also was no association between ATI and DGF. Only after inclusion of living donations into the analysis, higher grades of ATI caused more DGF (Supplementary Figure S1A). Though this surely only corresponds to the procedural differences between living and deceased donations.

In this cohort, there was no difference in death censored graft survival between transplants with and without DGF after 1 year. After the full observation period transplants without DGF had a significantly better survival (*p* = 0.045; Supplementary Figure S2A), suggesting an influence only on long-term graft survival.

ATI as well as IF/TA, glomerulosclerosis and arteriosclerosis did not influence 1-year eGFR after deceased donation. When including living donations, these results were no different, except ATI proving to show an association with eGFR, again likely caused by the procedural factors (Figure 1B; Supplementary Figure S1B). No associations between any histological parameters and proteinuria (mg/g creatinine), which was recorded up to 5 years after transplantation were found for deceased donations.

**FIGURE 2:**
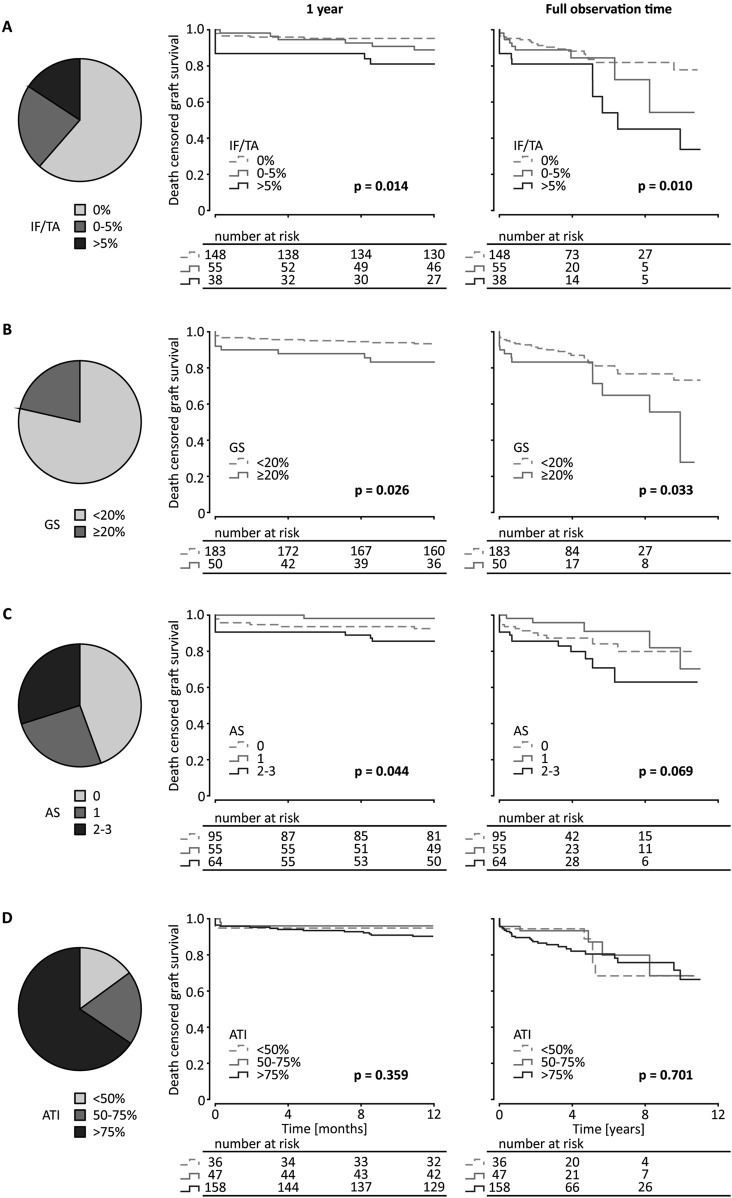
Kidney graft tissue was taken 10 min after reperfusion by 18G core needle biopsy. Histological evaluation was performed by one experienced renal pathologist. **(A)** semi-quantitative score according to the Banff Classification was used to assess arteriosclerosis (AS). Interstitial fibrosis and tubular atrophy (IF/TA), glomerulosclerosis (GS), and acute tubular injury (ATI) are shown as percentage of the entire area used for histological investigation. Only data from transplantation after deceased donation is included in this analysis. **(A)** Pie chart of the distribution of IF/TA in 3 categories (0%, 0%–5%, >5%). Kaplan-Meier estimates for short (1 year) and long term (11.4 years) death censored graft survival for the 3 IF/TA categories. **(B)** Pie chart of the distribution of GS in 2 categories (<20%, ≥20%) and Kaplan-Meier estimates for short- and long-term death censored graft survival for the 2 categories of GS. **(C)** Pie chart of the distribution of AS in 3 categories (grades 0, 1, 2–3) and Kaplan-Meier estimates for short- and long-term death censored graft survival for the 3 categories of arteriosclerosis. **(D)** Pie chart of the distribution of ATI in 3 categories (<50%, 50%–75%, >75%) and Kaplan-Meier estimates for short- and long-term death censored graft survival for the 3 ATI categories. Log-rank testing was used for calculation of each *p*-value.

For Kaplan-Meier analysis, ATI and arteriosclerosis were divided into groups as described above. IF/TA was analyzed in 3 groups as well: 0%, >0–5% and >5%. Glomerulosclerosis was analyzed as 2 groups: <20% and ≥20%. With lower degree of IF/TA short-term (1 year) and long-term (full follow-up period) death censored graft survival improved as shown in Kaplan-Meier analysis in deceased donations as well as living and deceased donations together. The same was observed for glomerulosclerosis. Arteriosclerosis only influenced short-term graft survival after deceased donation. ATI did not have any relevant influence on death censored graft survival for short- and long-term observation for deceased donations and the total cohort (Figure 2; Supplementary Figure S3). This was also the case, if only kidneys from ECD-donors were taken into consideration (Supplementary Figure S2B).

In univariable Cox proportional hazard analysis of transplantations after deceased donation, IF/TA showed a higher association than glomerulosclerosis with long-term graft survival (IF/TA: HR per 10% increase 1.63; 95% CI 1.17–2.28; *p* = 0.003; glomerulosclerosis: HR per 10% increase 1.19; 95% CI 1.01–1.39; *p* = 0.031). For short-time graft survival only IF/TA proved an association (IF/TA: HR per 10% increase 1.70; 95% CI 1.10–2.62; *p* = 0.016). ATI in baseline biopsies does not appear to be associated in univariable Cox proportional hazard models (Table 2). Arteriosclerosis grades 2 and 3 combined showed a significant HR compared with lower grades of arteriosclerosis only for long-term death censored graft survival after including living donations as well (HR 2.10; 95% CI 1.03–4.25; *p* = 0.040). IF/TA and glomerulosclerosis were also significantly associated with death censored graft survival when including all transplantations (Supplementary Table S1). Table 3 shows the hazard ratio for previously identified factors influencing kidney transplantation outcomes for long- and short-term death-censored graft survival.

**TABLE 2:** Univariable Cox proportional hazards models for 1-year and total-observation time for death censored graft survival of deceased donations with hazard ratios (HR) and 95% confidence intervals (CI) for post-reperfusion biopsy outcomes.

	1 year	*p*-value	Total observation time	*p*-value
IF/TA				
per 10% increase	1.70 (1.10–2.62)	**0.016**	1.63 (1.17–2.28)	**0.003**
Glomerulosclerosis				
per 10% increase	1.23 (0.98–1.56)	0.076	1.19 (1.01–1.39)	**0.031**
Arteriosclerosis				
grade 0 grade 1 grades 2 + 3	Reference 0.24 (0.03–1.96) 1.98 (0.74–5.32)	0.183 0.175	Reference 0.64 (0.23–1.79) 1.85 (0.87–3.96)	0.391 0.112
ATN				
0%–50% 51%–75% 76%–100%	Reference 0.76 (0.11–5.38) 1.86 (0.43–8.08)	0.781 0.409	Reference 0.76 (0.27–2.60) 1.19 (0.50–2.88)	0.757 0.692

*p*-values <0.05 are highlighted in bold.

**TABLE 3:** Univariable Cox proportional hazards models for 1-year and total-observation time for death censored graft survival with hazard ratios (HR) and 95% confidence intervals (CI) for donor, recipient and transplant associated factors.

	1 year	p-value	Total observation time	*p*-value
Donor associated				
Age	1.07 (1.03–1.11)	**<0.001**	1.05 (1.02–1.07)	**<0.001**
Gender (f)	0.88 (0.37–2.08)	0.766	0.90 (0.51–1.60)	0.729
BMI	1.03 (0.95–1.12)	0.440	1.01 (0.95–1.06)	0.853
ECD	4.74 (1.74–12.93)	**0.002**	3.24 (1.79–5.87)	**<0.001**
History of - hypertension - diabetes - smoking	3.48 (1.34–9.05) 3.50 (1.26–9.69) 0.40 (0.13–1.19)	**0.011** **0.016** 0.099	2.62 (1.45–4.75) 3.86 (1.95–7.65) 0.46 (0.22–0.97)	**0.012** **<0.001** **0.042**
Cause of death: CVA	2.85 (0.96–3.47)	**0.041**	1.91 (0.98–3.76)	0.059
last SCr	0.82 (0.49–8.60)	0.059	0.76 (0.42–1.20)	0.353
Recipient associated				
Age	1.06 (1.02–1.11)	**0.006**	1.03 (1.01–1.06)	**0.013**
BMI	1.06 (0.98–1.15)	0.158	1.07 (1.01–1.13)	**0.030**
Gender (f)	0.40 (0.14–1.20)	0.102	0.95 (0.53–1.71)	0.864
CCI	1.05 (0.72–1.52)	0.801	1.00 (0.78–1.29)	0.978
Reason for ESKD - glomerulonephritis - diabetes - hypertension	0.95 (0.37–2.45) 0.79 (0.18–3.37) 0.29 (0.04–2.13)	0.913 0.745 0.222	0.99 (0.53–1.85) 0.87 (0.34–2.19) 0.64 (0.25–1.61)	0.976 0.759 0.340
Duration of dialysis	1.01 (1.00–1.02)	0.320	1.01 (1.00–1.01)	0.086
Transplant associated				
Donation type deceased	8.07 (1.08–60.14)	**0.042**	2.05 (0.92–4.58)	0.081
CIT	1.04 (0.97–1.11)	0.270	1.02 (0.98–1.07)	0.260
Number of HLA-mismatches	1.71 (1.19–2.45)	**0.004**	1.35 (1.08–1.69)	**0.008**
PRA	1.01 (1.00–1.02)	0.230	1.01 (1.00–1.02)	**0.004**
DGF	1.65 (0.42–6.13)	0.458	1.91 (1.41–2.61)	**<0.001**
Number of BPR in first year	2.19 (1.55–3.10)	**<0.001**	1.80 (1.48–2.19)	**<0.001**
Number of all BPR			0.74 (0.49–1.11)	0.149

BMI, Body Mass Index; BPR, biopsy-proven rejection; CCI, Charlson Comorbidity Index; CIT, cold ischemia time; CVA, cerebro-vascular accident; DGF, delayed graft function; eGFR, estimated glomerular filtration rate; ECD, expanded criteria donor; ESKD, end stage kidney disease; HLA, Human leukocyte antigen; PRA, panel-reactive antibody; SCr, Serum creatinine; TX, transplantation.

*p*-values <0.05 are highlighted in bold.

In multivariable Cox regression models that included ECD-status, history of diabetes, number of human leukocyte antigen (HLA)-mismatches, or recipient age, none of the tested histological parameters showed a significant association with 1-year death-censored graft survival when including only deceased donations or all transplantations (Table 4; Supplementary Table S2, data for ATI and arteriosclerosis not shown). However, in a model focused on immunological co-variates with the number of HLA-mismatches, percentage of panel reactive antibodies, and ECD-status, IF/TA was significantly associated with long-term death-censored graft survival in deceased donations and the total cohort (deceased donation: HR 1.05; 95% CI 1.01–1.09; *p* = 0.007). IF/TA was also associated with long-term death-censored graft survival in models that included ECD-status, donor history of diabetes, and recipient age or number of HLA-mismatches in deceased donations (model including recipient age: HR 1.04; 95% CI 1.01–1.08; *p* = 0.023; model including HLA-mismatches: HR 1.04; 95%-CI 1.01–1.08; *p* = 0.022) as well as the total cohort. Glomerulosclerosis did not prove to be prognostic for long-term graft survival in any of the above-described models (Table 4; Supplementary Table S2).

**TABLE 4:** Multivariable Cox-regression model for 1-year and total-observation time for death censored graft survival of deceased donations with hazard ratios (HR) and 95% confidence intervals (CI) including prognostic factors for reduced graft survival.

Variables	1 year		1 year		max. follow-up		max. follow-up		max. follow-up	
	Model 1		Model 2		Model 1		Model 2		Model 3	
	HR (95% CI)	*p*-value	HR (95% CI)	*p*-value	HR (95% CI)	*p*-value	HR (95% CI)	*p*-value	HR (95% CI)	*p*-value
IF/TA	1.05 (0.99–1.11)	0.081	1.03 (0.99–1.09)	0.171	1.04 (1.01–1.08)	**0.022**	1.04 (1.01–1.08)	**0.023**	1.05 (1.01–1.09)	**0.007**
ECD	2.31 (0.72–7.34)	0.158	2.82 (0.82–9.72)	0.101	2.97 (1.41–6.29)	**0.004**	3.76 (1.72–8.23)	**<0.001**	3.03 (1.51–6.01)	**0.002**
Number HLA-miss matches	2.15 (1.35–3.43)	**0.001**			1.30 (0.99–1.72)	0.061			1.28 (0.98–1.67)	0.072
PRA									1.01 (1.00–1.02)	**0.005**
Recipient age			1.03 (0.98–1.09)	0.246			1.00 (0.97–1.03)	0.873		
h.o. diabetes	1.42 (0.48–4.12)	0.526	1.71 (0.69–4.87)	0.319	2.42 (1.18–5.00)	**0.016**	2.53 (1.23–5.20)	**0.012**		
Glomerulo-sclerosis					1.01 (0.99–1.03)	0.432	1.01 (0.99–1.03)	0.349	1.01 (1.00–1.03)	0.141
ECD					2.80 (1.32–5.95)	**0.007**	3.26 (1.50–7.01)	**0.003**	2.84 (1.41–5.72)	**0.001**
Number HLA-miss matches					1.28 (0.97–1.68)	0.086			1.24 (0.96–1.62)	0.101
PRA									1.01 (1.00–1.02)	**0.005**
Recipient age							1.00 (0.97–1.03)	0.851		
h.o. diabetes					2.56 (1.24–5.30)	**0.011**	2.66 (1.28–5.50)	**0.009**		

Models of 1 year graft survival including Glomerulosclerosis were neglected since univariable analysis showed no association. IF/TA, Interstitial fibrosis and tubular atrophy; HLA, Human leukocyte antigen; PRA, panel-reactive antibody.

*p*-values <0.05 are highlighted in bold.

No influence of IF/TA, glomerulosclerosis, arteriosclerosis, or ATI on the appearance of the first BPR was revealed by univariable Cox proportional hazard analysis (Supplementary Table S3).

### Influence of Immunological Parameters

As expected, the number of biopsy proven rejections during the first year after transplantation was highly associated with 1-year and long-term death censored graft survival (1 year: HR 2.19; 95% CI 1.55–3.08; *p* < 0.001; long-term: HR 1.80; 95% CI 1.48–2.19; *p* < 0.001). Interestingly, there was only a weak association between the number of HLA-mismatches and the number of biopsy proven rejections during the first year (r = 0.11; *p* = 0.042) which also persisted when including deceased donations only (r = 0–15; *p* = 0.024), and no association between percentage of PRA and BPR during the first year (r = 0.04; *p* = 0.51).

In a multivariable Cox-regression analysis with these 3 parameters, the number of BPR during the first year after transplantation and the number of HLA-mismatches were independently associated with 1-year death censored graft survival. In the same model, all 3 parameters were independently associated with long-term graft survival (Supplementary Table S4).

## Discussion

In this single-center retrospective study, we assessed the predictive value of post-transplant protocol biopsies conducted 10 min after onset of reperfusion, a standard practice in our transplant center. We leveraged a well-characterized cohort of kidney transplant recipients from both living and deceased donors. This allowed us to evaluate the relevance of histological findings against established clinical parameters encompassing donor and transplant characteristics, as well as immunological factors.

Studies investigating the influence of histological lesions in baseline biopsies on transplant success, DGF, and renal function have yielded heterogeneous results. A retrospective analysis found no association between ATI and DGF, acute rejection and graft survival in reperfusion biopsies [17]. Contrarily, increased risk for DGF was reported in donation after cardiac death (DCD) in grafts with reported ATI compared to no ATI [18]. Other data suggests reduced graft and recipient survival in severe chronic allograft injury in pre-transplant biopsies [19]. In another retrospective study glomerulosclerosis was the only histologic parameter associated with 5-year kidney allograft outcomes but did not outperform clinical parameters [20].

Complicating this narrative is the contentious backdrop of procurement biopsies. The intrinsic procedural demands accentuate cold ischemia times, with attendant augmentation in hemorrhage risks, as evidenced in a Portuguese study [21]. The interpretive acumen of histological assessments is also dependent on the expertise of the evaluating pathologist, with specialized pathologists delivering enhanced diagnostic insights compared to general pathologists [22].

In our study, all reviewed chronic parameters (IF/TA, glomerulosclerosis, arteriosclerosis) proved to have some association with death-censored graft survival in Kaplan-Meier analysis for short- and long-term observation in deceased donations only as well as the whole cohort. ATI solely did not offer any information about transplant survival. Opposing previous opinions, although arteriosclerosis showed a slight yet statistically significant association on renal transplant survival, it failed to achieve statistical significance when evaluated using Cox-regression, casting doubt on its actual influence [23, 24]. Given the intrinsic association between histological changes, we prioritized analysis of the correlation of each parameter individually rather than using a composite score.

The absence of an association between ATI and graft survival, corroborated by prior literature, challenges the notion that targeting ATI might enhance graft quality [2, 18, 25]. While ATI undeniably plays a pivotal role in DGF, and DGF is a recognized independent predictor of graft survival, our findings suggest that DGF’s impact operates independently of ATI, a finding recently confirmed by Wang et al. [26]. Instead, the repercussions of DGF may be more strongly influenced by organ quality metrics such as IF/TA and glomerulosclerosis. These metrics may heighten the graft’s vulnerability to ischemia-reperfusion injury. Ischemia-reperfusion injury, a principal driver for ATI, has been extensively researched in mouse models over recent years, primarily to identify therapeutic targets that bolster graft survival. However, none of these proposed targets have achieved clinical relevance so far [15, 27, 28]. Some authors argue in favor of interventions, especially for kidneys from marginal donors, to ameliorate ATI. Yet, our data does not support this perspective, particularly as ATI also did not correlate with graft survival even in ECD-grafts alone [29, 30].

Regardless of accumulating evidence challenging the utility of preimplantation biopsy findings, particularly due to the questionable predictive value from on-call pathologists lacking specialized renal pathology training, biopsy results still stand as the predominant reason for organ discard [31–33]. Our observation that only IF/TA demonstrates an association with long-term graft survival after adjusting for clinical parameters, in deceased donations only as well as the whole cohort, necessitates a strict reevaluation of the routinely employed procurement biopsies. Advocates for procurement or post-transplantation protocol biopsies often emphasize their potential in enabling personalized patient care, such as tailoring immunosuppression [34]. Indeed, the standalone association of IF/TA with long-term graft survival, coupled with the association with DGF based on conventional biopsy parameters, could bolster this argument. However, our data did not indicate a correlation between biopsy results and the occurrence of rejections. Consequently, the tangible additional insight offered by the biopsy appears limited. It’s conceivable that the inclusion of further immunologic histological parameters could enhance its value.

Our data confirmed the significance of established predictors for transplant survival after living and deceased donation. Next to donor history of diabetes, especially immunological parameters, meaning HLA-mismatches, PRA, and the number of biopsy proven rejections in the first year after transplantation proved to be strong and reliably associated with death censored graft survival in our cohort. Nonetheless, existing composite scores of these parameters fail to attain a concordance statistic above 0.7 [35, 36]. The mounting evidence favoring superior survival post-transplantation, compared to dialysis—even with organs deemed unsuitable for transplant, such as those labeled by the SCD/ECD classification—calls for strategies to avoid discarding potentially viable organs, particularly those of better quality [37–39]. In line with this, we found comparable 5-year graft and patient survival between standard and rescue allocation within our cohort which was previously published [40]. While histology provides valuable insights into organ quality without necessarily outperforming other parameters, we suggest that procurement biopsies could be particularly beneficial for organs typically overlooked. This notion warrants further exploration, as current guidelines for decision-making in this context are lacking.

This study warrants several critical discussions. We analyzed a single-center cohort comprising a moderate sample size, which included kidney transplants from both deceased and living donors. Without access to comparable data from other centers it what not possible to validate our findings against a different background. The differential selection processes and the potential variability in data availability between living and deceased donations may result in more detailed information for living donations. To reduce histological bias, the pathologist was entirely blinded to all patient-specific details. However, potential personal biases and biases by intra-observer variability might arise given that a single pathologist graded all biopsy samples. To decrease intra-observer variability the biopsies were not graded at the time of transplantation but at a single time point after collection of all samples. Previous data revealed insufficient diagnostic validity in histology performed by general pathologists, thus a highly specialized and experienced renal pathologist participated in this analysis [31]. The study’s follow-up lacked data on a substantial number of patients at the endpoints, possibly introducing a selection bias towards patients who were more adherent to their treatment regimens. Moreover, the inherent limitations of a retrospective design mean our study cannot achieve the rigor of a prospective observational study.

In conclusion, our findings support the persistent utility of established clinical and donor characteristics as primary predictors of kidney graft survival, with histological parameters playing a supplemental role [41]. Our findings indicate that while histological markers, specifically IF/TA, are associated with transplant outcomes, they do not surpass the predictive ability of established clinical indicators.

## Data Availability

The datasets presented in this article are not readily available because they were created for this retrospective analysis, thus we regard it as our intellectual property. It will be made available by the authors upon reasonable request. Requests to access the datasets should be directed to the corresponding author.

## Supplementary Material

DATA SHEET 1 Supplemental tables and supplemental figure legends.

IMAGE 1 Supplemental figure 1.

IMAGE 2 Supplemental figure 2.

IMAGE 3 Supplemental figure 3.

## Abbreviations

ATI, acute tubular injury; BPR, biopsy proven rejection; DCD, donation after cardiac death; DBD, donation after brainstem death; DGF, delayed graft function; ECD, expanded criteria donor; eGFR, estimated glomerular filtration rate; HR, hazard ratio; HLA, human leukocyte antigen; IF/TA, interstitial fibrosis and tubular atrophy; KDPI, kidney donor profile index; OR, odds ratio; SCD, standard criteria donor; PNF; primary non-function; PRA, panel-reactive antibodies.
