# Supplementary figures and images for: Only IF/TA in the Histological Evaluation of Post-Reperfusion Baseline Biopsies Correlates With Kidney Transplant Outcome
Source: Transpl Int. 2025 Jan 3;37:13646. doi: 10.3389/ti.2024.13646 (PMC11744053; doi:10.3389/ti.2024.13646)

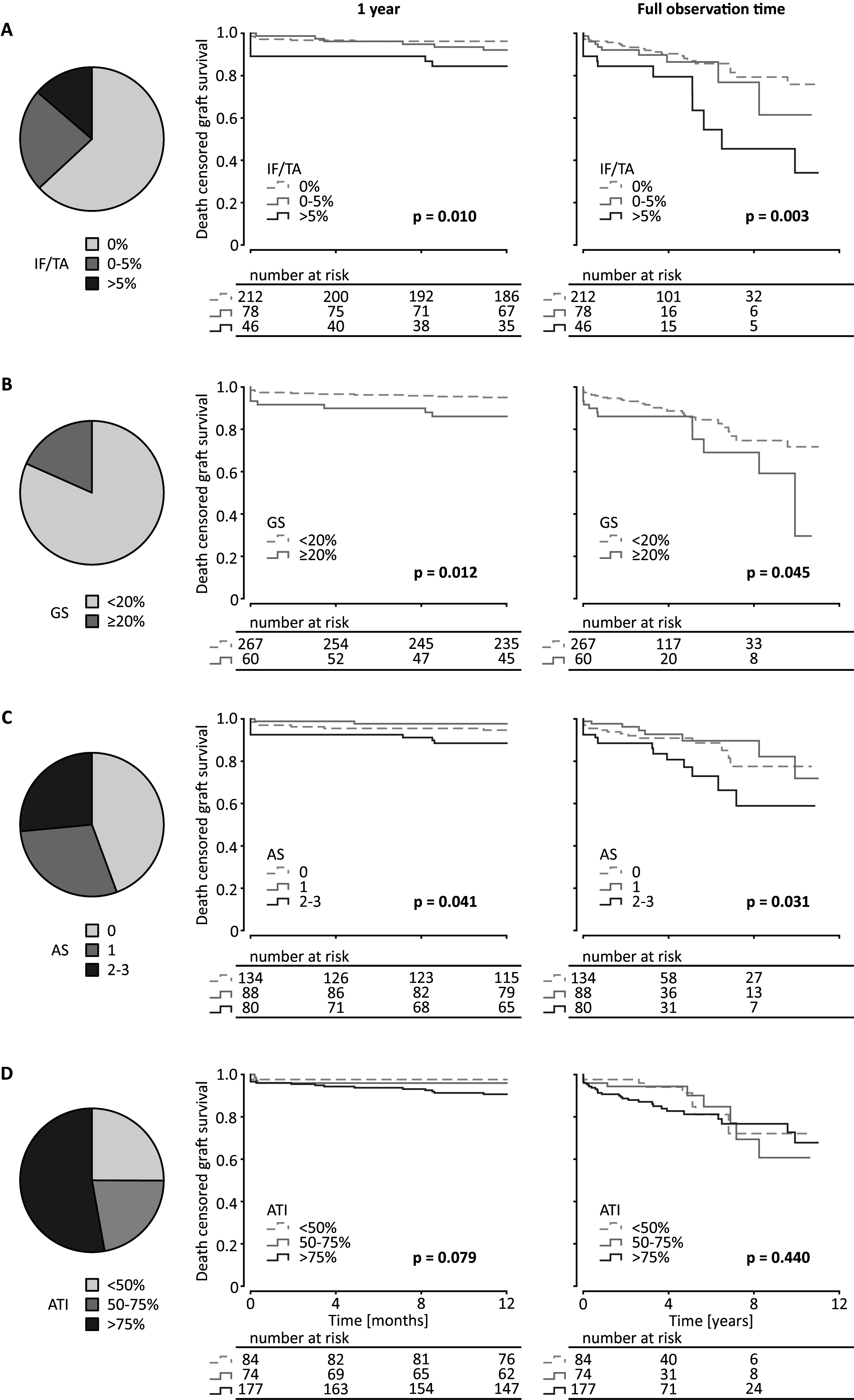

Supplement: Supplementary file 1 [file Image3.TIF]

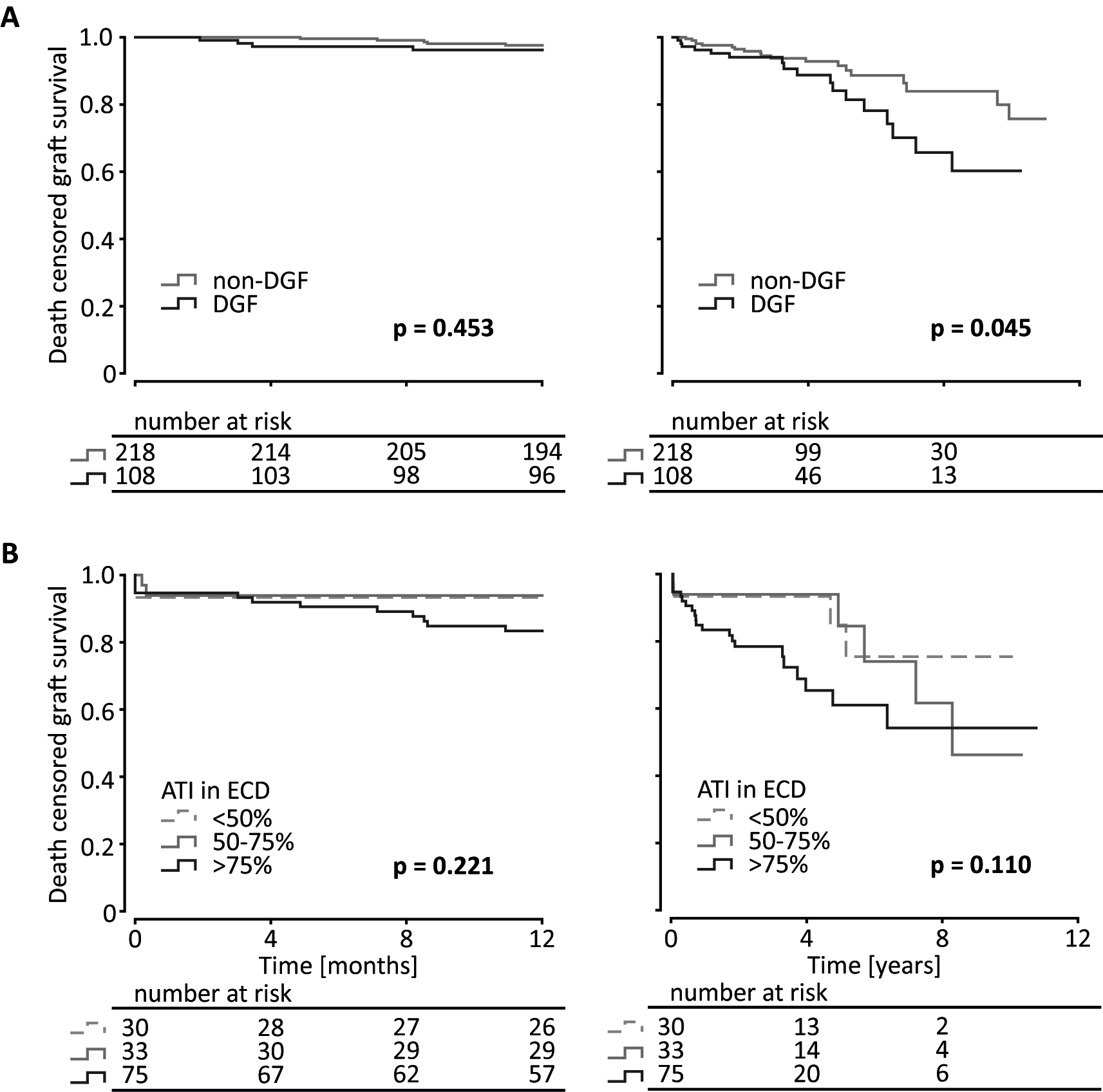

Supplement: Supplementary file 2 [file Image2.TIF]

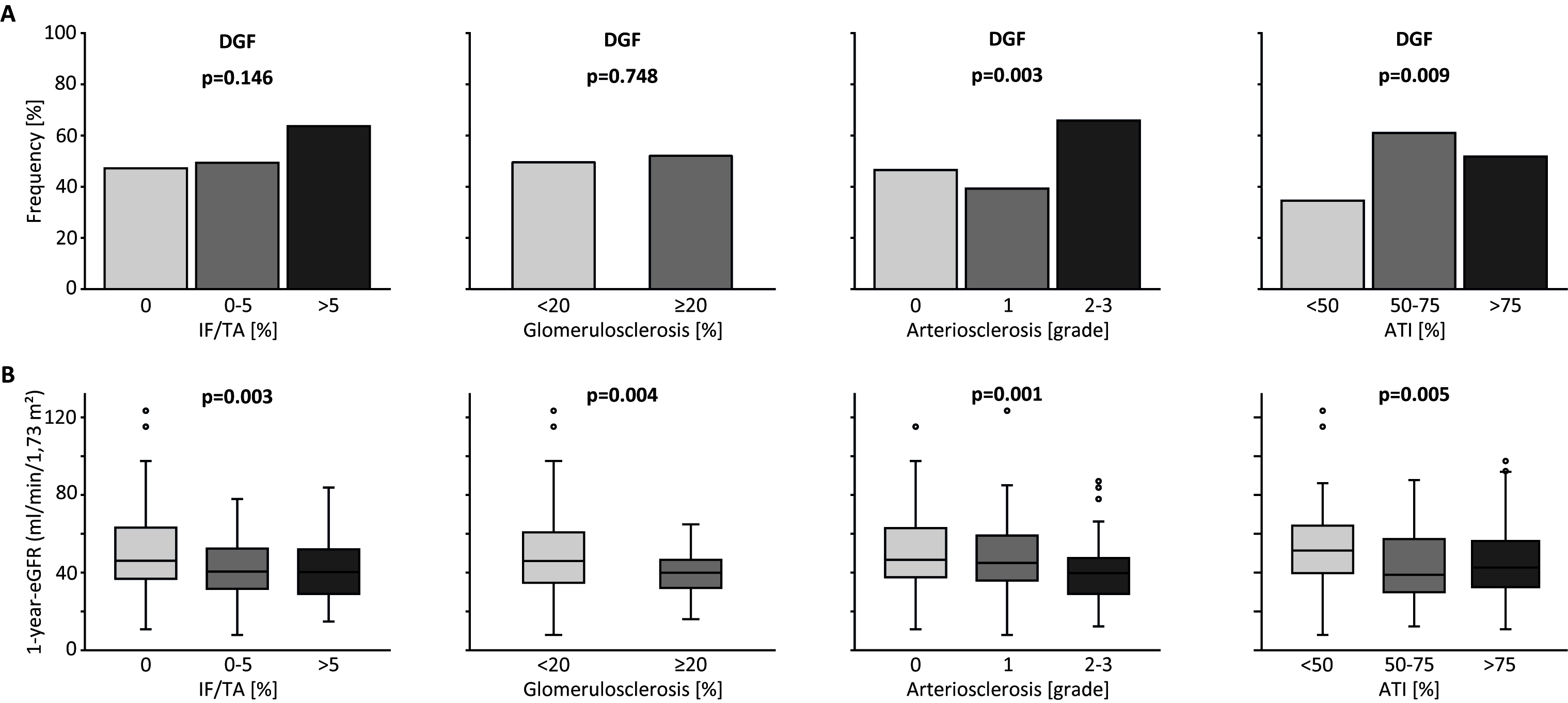

Supplement: Supplementary file 3 [file Image1.TIF]
