# Supplementary material for: Only IF/TA in the Histological Evaluation of Post-Reperfusion Baseline Biopsies Correlates With Kidney Transplant Outcome
Source: Transpl Int. 2025 Jan 3;37:13646. doi: 10.3389/ti.2024.13646 (PMC11744053; doi:10.3389/ti.2024.13646)
Supplement: Supplementary file 4 [file DataSheet1.docx]

**Only IF/TA in the histological evaluation of post-reperfusion baseline biopsies predicts kidney transplant outcome.**

**Supplemental material**

1. **TABLES**

***Supplemental table 1.*** *Univariable Cox proportional hazards models for one-year and total-observation time for death censored graft survival with hazard ratios (HR) and 95% confidence intervals (CI) for post-reperfusion biopsy outcomes. IF/TA: Interstitial fibrosis and tubular atrophy.*

|  | **1 year** | **p-value** | **total observation time** | **p-value** |
| --- | --- | --- | --- | --- |
| **IF/TA** | | |  |  |
| per 10% increase | 1.82 (1.18 - 2.80) | **0.007** | 1.70 (1.24 – 2.32) | **0.001** |
| **Glomerulosclerosis** | | |  |  |
| per 10% increase | 1.27 (1.01 - 1.61) | **0.036** | 1.19 (1.01 - 1.39) | **0.033** |
| **Arteriosclerosis** |  |  |  |  |
| grade 0  grade 1  grades 2+3 | reference  0.43 (0.09 - 2.06)  2.22 (0.83 - 5.97) | 0.290  0.113 | reference  0.80 (0.34 - 1.88)  2.10 (1.03 - 4.25) | 0.607  **0.040** |
| **ATN** |  |  |  |  |
| 0-50%  51-75%  76-100% | reference  1.75 (0.29 - 10.45)  3.94 (0.91 - 17.14) | 0.542  0.068 | reference  1.19 (0.47 - 3.00)  1.57 (0.74 - 3.32) | 0.715  0.238 |

**Supplemental table 2.** Multivariable *Cox-regression model for one-year and total-observation time for death censored graft survival with hazard ratios (HR) and 95% confidence intervals (CI) including prognostic factors for reduced graft survival. IF/TA: Interstitial fibrosis and tubular atrophy; HLA: Human leukocyte antigen; PRA: panel-reactive antibody.*

|  | **1 year** | | **1 year** | | **max. follow-up** | | **max. follow-up** | | **max. follow-up** | |
| --- | --- | --- | --- | --- | --- | --- | --- | --- | --- | --- |
| **Variables** | **model 1** | | **model 2** | | **model 1** | | **model 2** | | **model 3** | |
|  | **HR (95% CI)** | **p-value** | **HR (95% CI)** | **p-value** | **HR (95% CI)** | **p-value** | **HR (95% CI)** | **p-value** | **HR (95% CI)** | **p-value** |
| IF/TA | 1.04  (0.99 - 1.10) | 0.093 | 1.04  (0.99 - 1.09) | 0.146 | 1.04  (1.01 - 1.08) | **0.025** | 1.04  (1.01 - 1.07) | **0.026** | 1.05  (1.01 - 1.08) | **0.012** |
| ECD | 3.47  (1.11 - 10.87) | **0.032** | 3.44  (1.03 - 11.53) | **0.045** | 2.75  (1.41 - 5.34) | **0.003** | 2.92  (1.47 - 5.77) | **0.002** | 3.02  (1.61 - 5.65) | **<0.001** |
| Number HLA-miss matches | 1.76  (1.16 - 2.67) | **0.008** |  |  | 1.29  (1.01 - 1.64) | **0.042** |  |  | 1.27  (1.00 - 1.61) | **0.049** |
| PRA |  |  |  |  |  |  |  |  | 1.02  (1.01 - 1.02) | **<0.001** |
| Recipient age |  |  | 1.03  (0.98 - 1.08) | 0.247 |  |  | 1.01  (0.98 - 1.03) | 0.649 |  |  |
| h.o. diabetes | 1.87  (0.65 - 5.41) | 0.246 | 2.11  (0.74 - 6.00) | 0.163 | 2.88  (1.42 - 5.83) | **0.003** | 2.84  (1.41 - 5.71) | **0.004** |  |  |
| Glomerulo-  sclerosis | 1.015  (0.99 - 1.04) | 0.264 | 1.01  (0.99 - 1.04) | 0.343 | 1.01  (0.99 - 1.02) | 0.115 | 1.01  (0.99 - 1.02) | 0.469 | 1.01  (1.00 - 1.03) | 0.115 |
| ECD | 3.51  (1.12 - 10.96) | **0.031** | 3.24  (0.97 - 10.86) | 0.057 | 1.62  (1.34 - 5.10) | **0.005** | 2.65  (1.34 - 5.89) | **0.005** | 1.27  (1.00 - 1.60) | **0.001** |
| Number HLA-miss matches | 1.78  (1.17 - 2.72) | **0.008** |  |  | 1.27  (1.00 - 1.62) | 0.051 |  |  | 1.29  (1.0 - 1.64) | **0.042** |
| PRA |  |  |  |  |  |  |  |  | 1.02  (1.01 - 1.03) | **<0.001** |
| Recipient age |  |  | 1.03  (0.99 - 1.08) | 0.178 |  |  | 1.01  (0.98 - 1.04) | 0.442 |  |  |
| h.o. diabetes | 2.01  (0.71 - 5.71) | 0.191 | 2.21  (0.78 - 6.21) | 0.134 | 3.06  (1.49 - 6.30) | **0.002** | 3.05  (1.50 - 6.21) | **0.002** |  |  |

**Supplemental *table 3.*** *Univariable Cox proportional hazards models for biopsy proven rejections with one year after transplantation with hazard ratios (HR) and 95% confidence intervals (CI).*

|  | **1 year** | **p-value** |
| --- | --- | --- |
| **IF/TA** | | |
| per % increase | 1.01 (0.97 - 1.04) | 0.787 |
| **Glomerulosclerosis** | | |
| per % increase | 1.00 (0.98 - 1.02) | 0.959 |
| **Arteriosclerosis** |  |  |
| grade 0  grade 1  grades 2+3 | reference  0.66 (0.38 - 1.15)  0.82 (0.48 - 1.40) | 0.143  0.463 |
| **ATN** |  |  |
| 0-50%  51-75%  76-100% | reference  1.17 (0.66 - 2.09)  0.80 (0.48 - 1.33) | 0.588  0.389 |

**Supplemental table 4.** Multivariable *Cox-regression model for one-year and total-observation time for death censored graft survival with hazard ratios (HR) and 95% confidence intervals (CI) including immunological factors for graft survival. BPR: biopsy-proven rejection; HLA: Human leukocyte antigen; PRA: panel-reactive antibody.*

|  | **1 year** | | **max. follow-up** | | **1 year** | | **max. follow-up** | |
| --- | --- | --- | --- | --- | --- | --- | --- | --- |
| **Variables** | **death censored** | | **death censored** | | **non-death censored** | | **non-death censored** | |
|  | **HR (95% CI)** | **p-value** | **HR (95% CI)** | **p-value** | **HR (95% CI)** | **p-value** | **HR (95% CI)** | **p-value** |
| Number of BPRs during 1^st^ year | 2.28  (1.55 - 3.36) | **<0.001** | 1.92  (1.37 - 2.68) | **<0.001** | 1.89  (1.34 - 2.67) | **<0.001** | 1.66  (1.26 - 2.18) | **<0.001** |
| Number HLA-mis matches | 1.77  (1.24 - 2.53) | **0.002** | 1.43  (1.13 - 1.78) | **0.002** | 1.61  (1.22 - 2.14) | **<0.001** | 1.31  (1.11 - 1.55) | **0.001** |
| PRA | 1.01  (1.00 - 1.02) | 0.134 | 1.01  (1.00 - 1.02) | **0.004** | 1.01  (0.99 - 1.02) | 0.403 | 1.01  (1.00 - 1.01) | 0.181 |

**2 FIGURE LEGENDS**

**Supplemental figure 1:** Kidney graft tissue was taken 10 minutes after reperfusion by 18G core needle biopsy from living and deceased donations. Histological evaluation was performed by one experienced renal pathologist. A semi-quantitative score according to the Banff Classification was used to assess arteriosclerosis (AS). Interstitial fibrosis and tubular atrophy (IF/TA), glomerulosclerosis (GS), and acute tubular injury (ATI) are shown as percentage of the entire area used for histological investigation. **A)** Percent stacked column chart of the amount of delayed graft function (DGF) for different amounts of IF/TA, GS, AS, and ATI. **B)** Boxplots of the eGFR or kidney transplant recipients after living and deceased donation 1 year after transplantation. Groups are divided by histological categories as in A). Chi-squared test was used to compare categorial data. Kruskal-Wallis test was used for comparison of >2 groups with metric variables. Mann-Whitney U test was used for comparison of 2 groups with metric variables.

**Supplemental figure 2:** **A)** Kaplan-Meier estimates for short- and long-term death censored graft survival for patients with and without delayed graft function (DGF) after kidney transplantation from living and deceased donation. **B)** Kaplan-Meier estimates for short- and long-term death censored graft survival for the acute tubular injury (ATI) in 3 categories (<50%, 50-75%, >75%). Kidney graft tissue was taken 10 minutes after reperfusion by 18G core needle biopsy. Histological evaluation was performed by one experienced renal pathologist. Acute tubular injury is shown as percentage of the entire area used for histological investigation. Log-rank testing was used for calculation of each p-value.

**Supplemental figure 3:** Kidney graft tissue was taken 10 minutes after reperfusion by 18G core needle biopsy from living and deceased donations. Histological evaluation was performed by one experienced renal pathologist. A semi-quantitative score according to the Banff Classification was used to assess arteriosclerosis (AS). Interstitial fibrosis and tubular atrophy (IF/TA), glomerulosclerosis (GS), and acute tubular injury (ATI) are shown as percentage of the entire area used for histological investigation. **A)** Pie chart of the distribution of IF/TA in 3 categories (0%, 0-5%, >5%). Kaplan-Meier estimates for short (1 year) and long term (11.4 years) death censored graft survival for the 3 IF/TA categories. **B)** Pie chart of the distribution of GS in 2 categories (<20%, ≥20%) and Kaplan-Meier estimates for short- and long-term death censored graft survival for the 2 categories of GS. **C)** Pie chart of the distribution of AS in 3 categories (grades 0, 1, 2-3) and Kaplan-Meier estimates for short- and long-term death censored graft survival for the 3 categories of arteriosclerosis. **D)** Pie chart of the distribution of ATI in 3 categories (<50%, 50-75%, >75%) and Kaplan-Meier estimates for short- and long-term death censored graft survival for the 3 ATI categories. Log-rank testing was used for calculation of each p-value.
